# Gamma‐glutamyl transferase and the risk of all‐cause and disease‐specific mortality in patients with diabetes: A nationwide cohort study

**DOI:** 10.1111/1753-0407.13551

**Published:** 2024-04-25

**Authors:** Goh Eun Chung, Su‐Min Jeong, Su Jong Yu, Jeong‐Ju Yoo, Yuri Cho, Kyu‐na Lee, Dong Wook Shin, Yoon Jun Kim, Jung‐Hwan Yoon, Kyungdo Han, Eun Ju Cho

**Affiliations:** ^1^ Department of Internal Medicine and Healthcare Research Institute Seoul National University Hospital Healthcare System Gangnam Center Seoul Republic of Korea; ^2^ Department of Medicine Seoul National University College of Medicine Seoul Republic of Korea; ^3^ Department of Internal Medicine and Liver Research Institute Seoul National University College of Medicine Seoul Republic of Korea; ^4^ Department of Internal Medicine, Division of Gastroenterology and Hepatology Soonchunhyang University Bucheon Hospital Gyeonggi‐do Republic of Korea; ^5^ Center for Liver and Pancreatobiliary Cancer National Cancer Center Goyang Republic of Korea; ^6^ Department of Biomedicine & Health Science The Catholic University of Korea Seoul Korea; ^7^ Department of Clinical Research Design and Evaluation/Department of Digital Health Samsung Advanced Institute for Health Science Seoul Republic of Korea; ^8^ Department of Biostatistics College of Medicine, Soongsil University Seoul Republic of Korea

**Keywords:** diabetes mellitus, disease‐specific, gamma‐glutamyl transferase, mortality

## Abstract

**Background:**

There exists a paucity of data regarding whether gamma‐glutamyl transferase is associated with disease‐specific mortality in patients with type 2 diabetes mellitus. This study aimed to investigate the association of serum gamma‐glutamyl transferase levels with all‐cause and disease‐specific mortality in patients with diabetes mellitus using a Korean nationwide health‐screening database.

**Methods:**

A total of 9 687 066 patients without viral hepatitis or liver cirrhosis who underwent health examination in 2009 were included. These patients were divided into four groups according to sex‐specific quartiles of serum gamma‐glutamyl transferase levels.

**Results:**

During a median follow‐up period of 8.1 years, 222 242 deaths were identified. The all‐cause mortality rate increased as the serum gamma‐glutamyl transferase levels became higher (highest quartile vs lowest quartile: hazard ratio [HR], 1.57; 95% confidence interval [CI], 1.55–1.59; *p* for trend <.001). Similar trends were observed for cardiovascular disease (HR, 1.57; 95% CI, 1.53–1.62), ischemic heart disease (HR, 1.40; 95% CI, 1.33–1.48), and stroke (HR, 1.72; 95% CI, 1.60–1.85) in the highest quartile, as compared with the lowest quartile (*p* for trend <.001). As the gamma‐glutamyl transferase quartiles became higher, mortality rates related to cancer (HR, 1.56; 95% CI, 1.52–1.60), liver disease (HR, 9.42; 95% CI, 8.81–10.07), respiratory disease (HR, 1.55; 95% CI, 1.49–1.62), and infectious disease (HR, 1.73; 95% CI, 1.59–1.87) also increased in the highest quartile, compared with the lowest quartile (*p* for trend <.001).

**Conclusions:**

Serum gamma‐glutamyl transferase levels may be useful for the risk assessment of all‐cause and disease‐specific mortality among patients with type 2 diabetes mellitus.

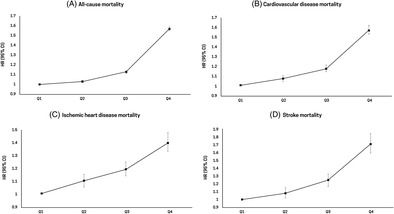

## Graphical abstract

Highlights

- This Korean nationwide population‐based cohort study analyzed the overall and disease‐specific mortality according to serum gamma‐glutamyl transferase (GGT) levels in patients with type 2 diabetes.
- GGT levels were positively associated with various causes of mortality, including cardiovascular disease, cancer, liver disease, respiratory disease, and infectious disease.
- Notably, the increased risk of all‐cause mortality was more prominent in the subgroups of the young, underweight, and those with complications of type 2 diabetes.
- Serum GGT levels may be useful for the risk assessment of all‐cause and disease‐specific mortality among patients with type 2 diabetes.

## 1 INTRODUCTION

Diabetes mellitus (DM) is a common chronic metabolic disease, with a global prevalence of approximately 10.5% in 2021 and with the majority of cases being type 2 DM (T2DM).^1^ An increase in all‐cause and disease‐specific mortality rates, including deaths related to cardiovascular disease (CVD), chronic respiratory disease, and kidney disease, has been reported in patients with DM.^2^, ^3^, ^4^ Thus, early detection of high‐risk patients is crucial for enhancing clinical management and providing timely intervention, which may lead to a reduction in the risk of premature death among patients with T2DM.

Gamma‐glutamyl transferase (GGT) is a ubiquitous enzyme responsible for extracellular glutathione catabolism and cellular antioxidant defense mechanisms.^5^ GGT has been regarded as a surrogate for oxidative stress and has been shown to play a role in the pathogenesis of various diseases, including CVD, cancer, and lung disease.^6^, ^7^, ^8^ In addition to its clinical utility as an indicator of hepatobiliary diseases and alcohol consumption, GGT has also been reported to be correlated with cardiometabolic risk factors, such as visceral adiposity, hepatic triglyceride accumulation, metabolic syndrome, and T2DM,^9^, ^10^, ^11^ and to be associated with a high risk of all‐cause and CVD‐specific mortality in both the general population^12^, ^13^, ^14^ and patients with T2DM.^15^, ^16^, ^17^ Nonetheless, except for CVD‐specific mortality, there exists a paucity of data regarding whether GGT is associated with disease‐specific mortality in patients with T2DM, as compared with those without diabetes.^7^ Furthermore, previous studies included only a relatively small number of participants, thereby restricting their detailed analysis of the relationship between GGT and disease‐specific mortality.^15^, ^16^, ^17^, ^18^ Additionally, some studies did not consider underlying chronic liver diseases, which might have obscured their results.^16^, ^17^, ^19^

Given the potential role of GGT in metabolic disease mechanisms, we hypothesized that GGT could serve as a simple and noninvasive prognostic tool for predicting mortality in patients with T2DM. Therefore, the present study aimed to investigate the association of serum GGT levels with all‐cause and disease‐specific mortality in patients with DM. In this study, we comprehensively analyzed the overall and disease‐specific mortality according to GGT levels in patients with T2DM and evaluated their prognostic implications based on a nationwide population‐based cohort in South Korea.

## 2 MATERIALS AND METHODS

### 2.1 Data source and study setting

The present study used data obtained from Korea's National Health Insurance System (NHIS) database. As a single government insurer, the NHIS provides a mandatory universal insurance system covering 97% of the entire Korean population, whereas the Medical Aid Program covers the remaining 3% of the population with low income. The NHIS collects information on insurance eligibility and medical utilization claims (history of medication prescription, diagnosis, etc.) and offers general health examination at least every 2 years for all insured individuals.^20^ The national health examination consists of an assessment of lifestyle habits (alcohol consumption, smoking, and physical activity) using a standard questionnaire; anthropometric measurements (height, weight, body mass index [BMI], waist circumference, and blood pressure); and laboratory tests. According to the 2013 NHIS report, 72.1% of eligible participants underwent the national health examination.^21^ The results of this national health examination are linked to the NHIS database, which has been widely used by epidemiological studies.^22^

### 2.2 Study population

Among individuals who underwent health screening from 2009 to 2012, a total of 2 745 689 patients with T2DM aged ≥20 years were identified. T2DM was identified by the presence of at least one of the following two criteria: (a) individuals with fasting plasma glucose levels ≥126 mg/dL on health examination (ie, undiagnosed DM) and (b) individuals who had at least one service claim with a diagnosis of T2DM (*International Classification of Diseases, Tenth Revision* [ICD‐10] codes E11–14) and were prescribed with antidiabetic agents prior to health examination. These operational definitions of T2DM based on population based NHIS claims data, including diagnostic and prescription codes, had been previously validated in the Korean population.^23^ Subsequently, patients with hepatitis (ICD‐10 codes B15–18; *n* = 417 798), patients with liver cirrhosis (ICD‐10 code K74; *n* = 19 645), and heavy drinkers who drank ≥30 g/day (for men) or ≥ 20 g/day (for women) of alcohol (*n* = 188 879) were excluded. Additionally, patients with missing data (*n* = 93 978, Table S1) and those who died within 1 year after the health examination (*n* = 17 614) were excluded to reduce the effect of reverse causality (1‐year lag time). Finally, 2 007 775 patients with T2DM were included in this study.

This study was conducted in accordance with the principles embodied in the Declaration of Helsinki, and all procedures were performed in compliance with relevant laws and institutional guidelines. Furthermore, this study was approved by the Institutional Review Board (IRB) of Soongsil University (IRB approval no: SSU‐202003‐HR‐201‐01). The requirement for the acquisition of written informed consent from participants was waived owing to the use of anonymous and de‐identified information.

### 2.3 Laboratory findings

Serum GGT, aspartate aminotransferase, alanine transaminase (ALT), and glucose levels, as well as lipid profiles, were measured on the day of health examination using a Hitachi Automatic Analyzer 7600 (Hitachi, Tokyo, Japan).^24^ Serum GGT levels were categorized into the following sex‐specific quartiles: lowest quartile (<25 IU/L for men and < 16 IU/L for women), second quartile (25–38 IU/L for men and 16–22 IU/L for women), third quartile (39–65 IU/L for men and 23–34 IU/L for women), and highest quartile (≥66 IU/L for men and ≥ 35 IU/L for women).

### 2.4 Outcomes

The cohort was followed up from 1 year after the date of health examination to the date of death or until the end of the study period (December 31, 2018), whichever came first. Data on the date and cause of death, which were linked to the NHIS database, were provided by Statistics Korea based on death certificates. The cause of death was identified as follows using the Korean Standard Classification of Diseases and Causes of Death in accordance with the following ICD‐10 codes: CVD (I00–99), including ischemic heart disease (I20–25) and ischemic stroke (I63–64); cancer (C00–97), excluding hepatocellular carcinoma (C22); liver disease (C22, K70–76); respiratory disease (J00–99); infectious disease (A00‐B99); and T2DM (E11–14).^25^, ^26^

### 2.5 Covariates

BMI was calculated as weight (kg) divided by height squared (m^2^). Lifestyle habits were assessed using a self‐reported questionnaire. Smoking status was categorized into nonsmoker, former smoker, and current smoker. Alcohol consumption was categorized according to the daily amount of consumed alcohol into nondrinker and mild‐to‐moderate drinker (<30 g/day for men and < 20 g/day for women). Regular physical activity was defined as engaging in regular high‐intensity exercise three times or more per week or in regular moderate‐intensity exercise five times or more per week. Insurance premium levels were used to determine the income levels and were categorized into quartiles, with those covered by the Medical Aid Program (3% of the poorest) being merged into the lowest income quartile.

The presence of comorbidities, including hypertension and dyslipidemia, was defined using a combination of clinical and claims data (ICD‐10 codes with prescription of relevant medications). In particular, hypertension was defined as elevated blood pressure (systolic blood pressure ≥ 140 mm Hg or diastolic blood pressure ≥ 90 mm Hg) or ICD‐10 codes I10–13 and I15 with prescription of antihypertensive medications, whereas dyslipidemia was defined as total cholesterol level ≥ 240 mg/dL or ICD‐10 code E78 with prescription of lipid‐lowering medications. A history of cancer was defined as having both ICD‐10 codes C00–99 and records of rare incurable diseases due to cancer; patients with cancer could be registered for benefit extension. The Charlson Comorbidity Index was calculated using the ICD‐10 codes.^27^

The presence of T2DM complications was defined as having at least one of the following complications: diabetic retinopathy (H36.0), end‐stage renal disease (registration records of rare incurable disease), stroke (I63–64 with brain images during hospitalization), and ischemic heart disease (I20–25). T2DM duration was categorized into newly diagnosed (T2DM detected at the time of health examination), <5 years, and ≥5 years based on claim history (time from first appearance of ICD‐10 codes with antidiabetic agents to the day of health examination). T2DM status was evaluated using the number of oral hypoglycemic agents (1 or ≥2) and insulin use.

### 2.6 Statistical analysis

Continuous and categorical variables are expressed as means ± SDs and as number (%), respectively. The number of deaths and the all‐cause and disease‐specific mortality rates per 1000 person‐years were assessed according to sex‐specific quartiles. The association between GGT quartiles and mortality in patients with T2DM was examined using Cox proportional hazards regression analysis. Model 1 was adjusted for age and sex, whereas model 2 was adjusted for age, sex, BMI, income levels, serum ALT levels, lifestyle factors (smoking status, alcohol consumption, and physical activity), hypertension, dyslipidemia, and Charlson Comorbidity Index. In addition to the covariates in model 2, model 3 was also adjusted for the presence of T2DM complications and the T2DM duration. Stratified analysis was conducted according to sex, age, BMI, and presence of T2DM complications. Statistical analyses were performed using SAS version 9.4 (SAS Institute Inc., Cary, NC, USA), with statistical significance set a *p* value of <.05.

## 3 RESULTS

### 3.1 Baseline characteristics

Table 1 summarizes the baseline characteristics of the study population according to GGT quartiles. The mean age became younger as the GGT quartiles became higher (59.9 and 55.2 years in the lowest and highest quartiles, respectively). The participants in the lowest GGT quartile were more likely to be less obese, nonsmokers, and nondrinkers and to engage more in regular exercise than those in other quartiles. A higher proportion of comorbidities was detected among participants in the highest GGT quartile. T2DM was more severe among participants in the lowest GGT quartile. In particular, the rates of T2DM complications, T2DM duration ≥5 years, oral hypoglycemia agent use ≥2, and insulin use were higher among participants in the lowest GGT quartile.

**TABLE 1:** Baseline characteristics of study population by quartiles of gamma‐glutamyl transferase.

	Gamma‐glutamyl transferase			
	Q1 men: < 25 IU/L and women: < 16 IU/L	Q2 men: 25–38 IU/L and women: 16–22 IU/L	Q3 men: 39–65 IU/L and women: 23–34 IU/L	Q4 men: ≥ 66 IU/L and women: ≥ 35 IU/L
Number	506 252	494 164	508 207	499 152
Age (years)	59.9 ± 13.3	58.8 ± 12.2	57.3 ± 11.9	55.2 ± 11.8
Sex*, n* (%)				
Male	280 730 (55.5)	294 293 (59.6)	284 197 (55.9)	286 705 (57.4)
Female	225 522 (44.6)	199 871 (40.5)	224 010 (44.1)	212 447 (42.6)
Smoking*, n* (%)				
Non	325 149 (64.2)	289 709 (58.6)	295 307 (58.1)	270 918 (54.3)
Former	91 109 (18.0)	94 180 (19.1)	87 332 (17.2)	79 801 (16.0)
Current	89 994 (17.8)	110 275 (22.3)	125 568 (24.7)	148 433 (29.7)
Drinking*, n* (%)				
Non	374 614 (74.0)	322 407 (65.2)	302 818 (59.6)	247 196 (49.5)
Mild to moderate	131 638 (26.0)	171 757 (34.8)	205 389 (40.4)	251 956 (50.5)
Regular exercise*, n* (%)	115 083 (22.7)	106 640 (21.6)	99 147 (19.5)	88 848 (17.8)
Income, lowest Q1*, n* (%)	107 162 (21.2)	104 017 (21.1)	107 320 (21.1)	108 971 (21.8)
Comorbidities*, n* (%)				
Hypertension	260 800 (51.5)	275 945 (55.8)	296 072 (58.3)	298 628 (59.8)
Dyslipidemia	176 032 (34.8)	200 848 (40.6)	229 263 (45.1)	241 337 (48.4)
Cancer	17 475 (3.5)	14 155 (2.9)	12 473 (2.5)	10 828 (2.2)
CCI score	2.5 ± 2.1	2.4 ± 2.1	2.2 ± 2.0	2.0 ± 2.0
T2DM complication*, n* (%)	122 364 (24.2)	105 978 (21.5)	96 207 (18.9)	77 417 (15.5)
Retinopathy	66 993 (13.2)	50 516 (10.2)	41 370 (8.1)	29 906 (6.0)
ESRD	2269 (0.5)	1174 (0.2)	922 (0.2)	659 (0.1)
Stroke	3141 (0.6)	2914 (0.6)	2849 (0.6)	2350 (0.5)
IHD	66 764 (13.2)	64 094 (13.0)	61 824 (12.2)	52 271 (10.5)
T2DM duration*, n* (%)				
New‐onset	160 132 (31.6)	170 287 (34.5)	194 829 (38.3)	225 971 (45.3)
< 5 years	140 198 (27.7)	154 395 (31.2)	169 618 (33.4)	166 327 (33.3)
≥ 5 years	205 922 (40.7)	169 482 (34.3)	143 760 (28.3)	106 854 (21.4)
Number of OHA*, n* (%)				
0	179 662 (35.5)	186 021 (37.6)	208 282 (41.0)	237 324 (47.6)
1	238 867 (47.2)	232 321 (47.0)	230 257 (45.3)	203 006 (40.7)
≥2	87 723 (17.3)	75 822 (15.3)	69 668 (13.7)	58 822 (11.8)
Insulin use, *n* (%)	55 820 (11.0)	43 167 (8.7)	37 074 (7.3)	32 707 (6.6)
BMI, kg/m^2^	23.7 ± 3.1	24.8 ± 3.1	25.6 ± 3.3	26.1 ± 3.6
WC, cm	82.0 ± 8.4	84.8 ± 8.3	86.4 ± 8.4	87.4 ± 8.6
Systolic BP, mm Hg	126.3 ± 15.8	128.4 ± 15.6	129.7 ± 15.6	131.2 ± 16.0
Diastolic BP, mm Hg	76.5 ± 9.9	78.3 ± 9.9	79.6 ± 10.1	81.1 ± 10.5
Laboratory test				
Glucose, mg/dL	137.3 ± 44.9	142.7 ± 46.5	146.6 ± 47.0	152.0 ± 48.0
Total cholesterol, mg/dL	184.6 ± 38.3	194.4 ± 40.2	201.6 ± 42.1	208.4 ± 45.8
Triglyceride, mg/dL*	128.8 ± 84.3	157.1 ± 106.9	182.4 ± 124.2	219.2 ± 166.5
HDL‐C, mg/dL	51.7 ± 24.0	51.3 ± 24.5	51.4 ± 22.5	52.8 ± 23.2
LDL‐C, mg/dL	108.2 ± 37.0	113.1 ± 39.4	115.2 ± 41.3	113.8 ± 44.9
AST, IU/L*	20.7 (20.7–20.7)	22.6 (22.6–22.6)	25.4 (25.3–25.4)	33.9 (33.9–34.0)
ALT, IU/L*	17.9 (17.8–17.9)	22.2 (22.2–22.3)	27.2 (27.2–27.3)	38.4 (38.4–38.5)
GGT, IU/L*	15.3 (15.3–15.3)	25.5 (25.5–25.5)	38.1 (38.0–38.1)	86.1 (86.0–86.3)

*Note*: Values are presented as number (%) or mean ± standard deviation. All *p*‐value <0.001.

Abbreviations: ALT, alanine transaminase; AST, aspartate aminotransferase; BMI, body mass index; BP, blood pressure; CCI, Charlson Comorbidity Index; ESRD, end stage renal disease; GGT, gamma‐glutamyl transferase; HDL‐C, high‐density lipoprotein‐cholesterol; IHD, ischemic heart disease; LDL‐C, low‐density lipoprotein‐cholesterol; OHA, oral hypoglycemic agent; T2DM, type 2 diabetes mellitus; WC, waist circumference.

* Geometric mean (95% confidence interval).

### 3.2 Association of GGT quartiles with all‐cause and CVD‐specific mortality

**FIGURE 1:**
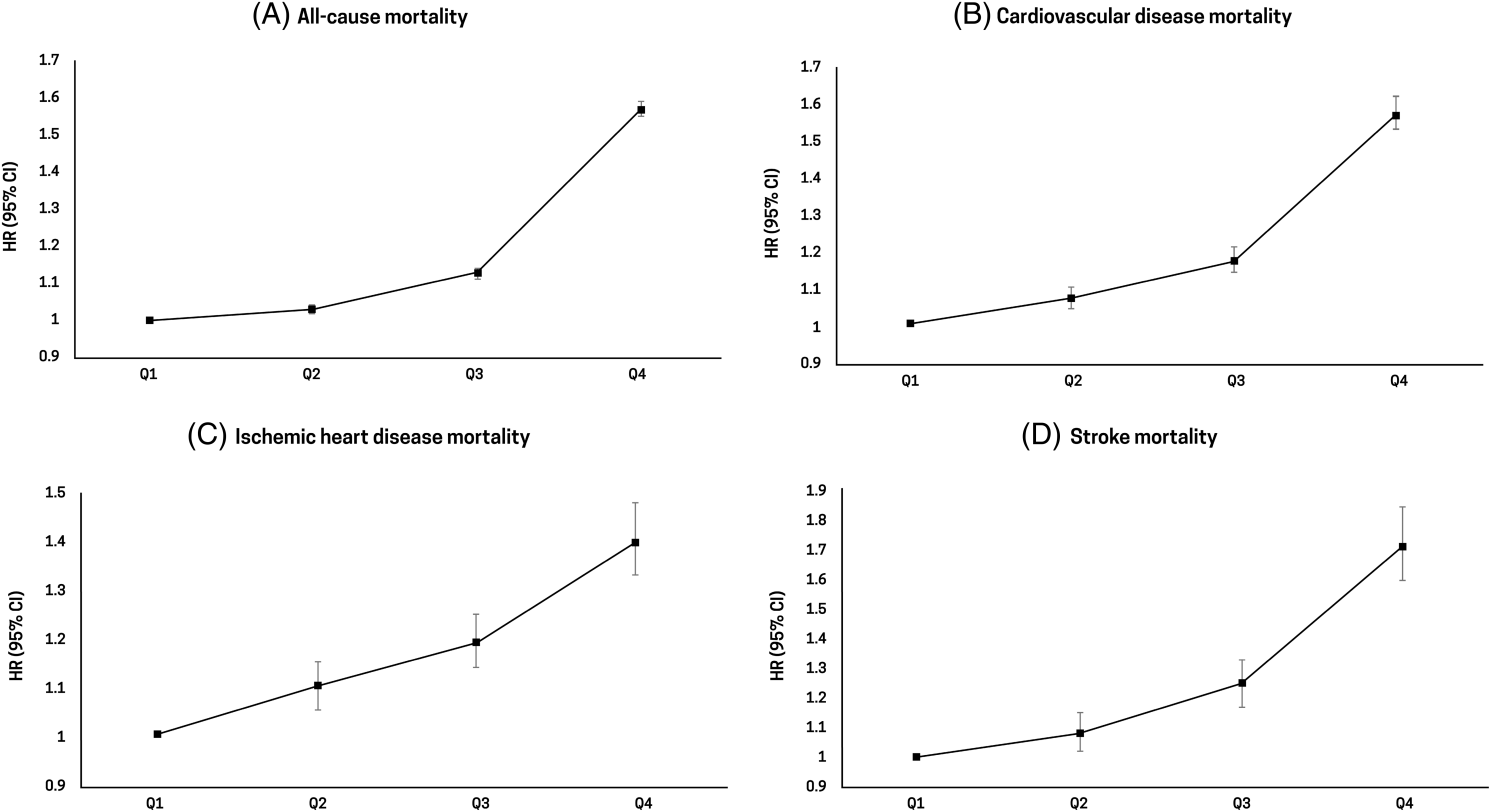
Hazard ratios (95% confidence intervals) for (A) all‐cause, (B) cardiovascular disease, (C) ischemic heart disease, and (D) stroke mortality according to quartiles of serum gamma‐glutamyl transferase level. CI, confidence interval; HR, hazard ratio.

During a median follow‐up period of 8.1 years, 222 242 deaths were identified in the total population, with a mortality rate of 14.3 per 1000 person‐years. The risk of all‐cause mortality increased as the GGT quartiles became higher (highest GGT quartile vs lowest GGT quartile: hazard ratio [HR], 1.57; 95% confidence interval [CI], 1.55–1.59; *p* for trend <.001) (Table 2). Similarly, the risk of CVD‐specific mortality increased as the GGT quartiles became higher (highest GGT quartile vs lowest GGT quartile: HR, 1.57; 95% CI, 1.53–1.62; *p* for trend <.001). The highest mortality rates for ischemic heart disease (HR, 1.40; 95% CI, 1.33–1.48) and stroke (HR, 1.72; 95% CI, 1.60–1.85) were observed in the highest GGT quartile (Figure 1). Similar associations were observed in both male and female participants; however, increased all‐cause and CVD‐specific mortality rates in male participants were more pronounced than those in female participants (Table S2). When we performed sensitivity analysis excluding participants with a history of CVD or cancer, the increased risk of all‐cause mortality according to the higher GGT quartile remained (Table S3).

**TABLE 2:** The association between gamma‐glutamyl transferase quartiles and all‐cause and cardiovascular disease‐specific mortality.

	Number	No. of events	Duration	IR, per 1000	Model 1	Model 2	Model 3
					HR (95% CI)	HR (95% CI)	HR (95% CI)
All‐cause mortality							
Q1	506 252	69 555	3896307.3	17.9	1 (Ref.)	1 (Ref.)	1 (Ref.)
Q2	494 164	55 225	3855130.1	14.3	0.94 (0.93–0.95)	1.02 (1.01–1.03)	1.03 (1.02–1.04)
Q3	508 207	48 094	3982949.6	12.1	0.97 (0.96–0.98)	1.10 (1.09–1.12)	1.13 (1.11–1.14)
Q4	499 152	49 368	3860408.1	12.8	1.29 (1.27–1.30)	1.52 (1.50–1.54)	1.57 (1.55–1.59)
CVD‐specific mortality							
Q1	506 252	14 943	3896307.3	3.8	1 (Ref.)	1 (Ref.)	1 (Ref.)
Q2	494 164	12 188	3855130.1	3.2	0.99 (0.96–1.01)	1.05 (1.03–1.08)	1.07 (1.04–1.10)
Q3	508 207	10 591	3982949.6	2.7	1.01 (0.99–1.04)	1.14 (1.11–1.17)	1.17 (1.14–1.21)
Q4	499 152	9846	3860408.1	2.6	1.23 (1.20–1.26)	1.51 (1.46–1.55)	1.57 (1.53–1.62)
Ischemic heart disease mortality							
Q1	506 252	4249	3896307.3	1.1	1 (Ref.)	1 (Ref.)	1 (Ref.)
Q2	494 164	3652	3855130.1	0.9	1.01 (0.97–1.06)	1.07 (1.02–1.12)	1.10 (1.05–1.15)
Q3	508 207	3124	3982949.6	0.8	1.02 (0.97–1.07)	1.13 (1.08–1.19)	1.19 (1.14–1.25)
Q4	499 152	2583	3860408.1	0.7	1.08 (1.02–1.13)	1.29 (1.22–1.37)	1.40 (1.33–1.48)
Stroke mortality							
Q1	506 252	2322	3896307.3	0.6	1 (Ref.)	1 (Ref.)	1 (Ref.)
Q2	494 164	1818	3855130.1	0.5	0.99 (0.93–1.05)	1.07 (1.00–1.14)	1.08 (1.02–1.15)
Q3	508 207	1596	3982949.6	0.4	1.05 (0.99–1.12)	1.22 (1.14–1.31)	1.25 (1.17–1.33)
Q4	499 152	1477	3860408.1	0.4	1.32 (1.23–1.41)	1.67 (1.55–1.80)	1.72 (1.60–1.85)

*Note*: Model 1 was adjusted for age and sex and Model 2 was adjusted for age, sex, body mass index, income levels, serum alanine transaminase levels, lifestyle factors (smoking status, alcohol consumption, physical activity), hypertension, dyslipidemia, and Charlson Comorbidity Index score. Model 3 was additionally adjusted for presence of T2DM complications and duration of T2DM to covariates in model 2.

Abbreviations: CI, confidence interval; CVD, cardiovascular disease; HR, hazard ratio; IR, incidence rate per 1000 person years; T2DM, type 2 diabetes mellitus.

### 3.3 Association between GGT quartiles and disease‐specific mortality

**FIGURE 2:**
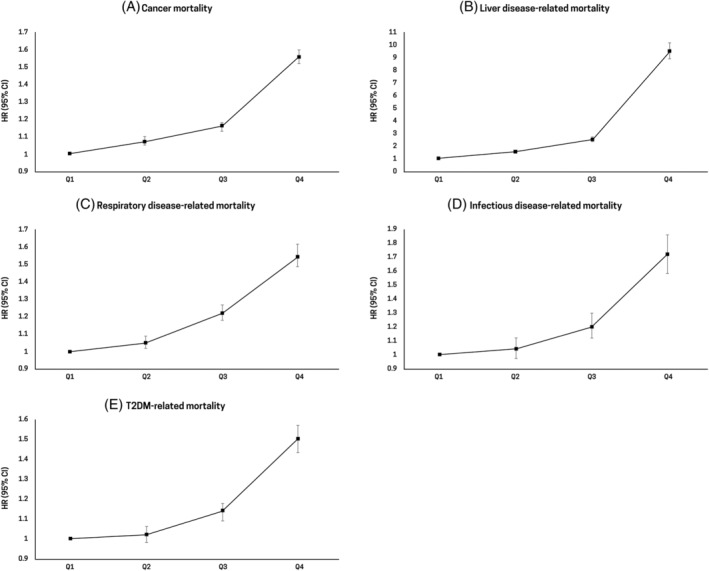
Hazard ratios (95% confidence intervals) for (A) cancer‐, (B) liver disease‐related (C) respiratory disease‐related (D) infection disease‐related‐, and (E) T2DM‐related mortality according to quartiles of serum gamma‐glutamyl transferase level. CI, confidence interval; HR, hazard ratio; T2DM, type 2 diabetes mellitus.

Table 3 and Figure 2 show the disease‐specific mortality according to GGT quartiles. Mortality rates related to cancer (HR, 1.56; 95% CI, 1.52–1.60), liver disease (HR, 9.42; 95% CI, 8.81–10.07), respiratory disease (HR, 1.55; 95% CI, 1.49–1.62), infectious disease (HR, 1.73; 95% CI, 1.59–1.87), and T2DM (HR, 1.51; 95% CI, 1.44–1.58) increased as the GGT quartiles became higher (highest GGT quartile vs lowest GGT quartile: *p* for trend <.001).

**TABLE 3:** The association between gamma‐glutamyl transferase quartiles and all‐cause mortality.

	Number	No. of events	Duration	IR, per 1000	Model 1	Model 2	Model 3
					HR (95% CI)	HR (95% CI)	HR (95% CI)
Cancer mortality							
Q1	506 252	18 225	3896307.3	4.7	1 (Ref.)	1 (Ref.)	1 (Ref.)
Q2	494 164	16 412	3855130.1	4.3	1.04 (1.02–1.06)	1.07 (1.05–1.10)	1.07 (1.05–1.10)
Q3	508 207	14 786	3982949.6	3.7	1.10 (1.08–1.13)	1.16 (1.13–1.18)	1.16 (1.13–1.18)
Q4	499 152	15 911	3860408.1	4.1	1.49 (1.45–1.52)	1.57 (1.53–1.60)	1.56 (1.52–1.60)
Liver disease‐related mortality							
Q1	506 252	1162	3896307.3	0.3	1 (Ref.)	1 (Ref.)	1 (Ref.)
Q2	494 164	1446	3855130.1	0.4	1.42 (1.31–1.53)	1.51 (1.40–1.63)	1.52 (1.41–1.65)
Q3	508 207	1910	3982949.6	0.5	2.23 (2.07–2.40)	2.44 (2.27–2.63)	2.48 (2.31–2.68)
Q4	499 152	6089	3860408.1	1.6	8.81 (8.26–9.39)	9.18 (8.59–9.81)	9.42 (8.81–10.07)
Respiratory disease‐related mortality							
Q1	506 252	8086	3896307.3	2.1	1 (Ref.)	1 (Ref.)	1 (Ref.)
Q2	494 164	5540	3855130.1	1.4	0.88 (0.85–0.91)	1.05 (1.01–1.08)	1.05 (1.02–1.09)
Q3	508 207	4467	3982949.6	1.1	0.91 (0.88–0.95)	1.21 (1.17–1.26)	1.22 (1.18–1.27)
Q4	499 152	3641	3860408.1	0.9	1.04 (0.99–1.08)	1.53 (1.46–1.60)	1.55 (1.49–1.62)
Infectious disease‐related mortality							
Q1	506 252	1871	3896307.3	0.5	1 (Ref.)	1 (Ref.)	1 (Ref.)
Q2	494 164	1417	3855130.1	0.4	0.92 (0.86–0.98)	1.03 (0.96–1.10)	1.04 (0.97–1.12)
Q3	508 207	1283	3982949.6	0.3	0.97 (0.91–1.05)	1.17 (1.09–1.26)	1.20 (1.12–1.30)
Q4	499 152	1326	3860408.1	0.3	1.33 (1.24–1.43)	1.66 (1.54–1.80)	1.73 (1.59–1.87)
T2DM‐related mortality							
Q1	506 252	7447	3896307.25	1.9	1 (Ref.)	1 (Ref.)	1 (Ref.)
Q2	494 164	5260	3855130.06	1.4	0.84 (0.81–0.87)	0.96 (0.92–0.99)	1.02 (0.98–1.06)
Q3	508 207	4291	3982949.58	1.1	0.80 (0.77–0.83)	1.01 (0.97–1.05)	1.14 (1.09–1.18)
Q4	499 152	3688	3860408.1	1.0	0.89 (0.85–0.93)	1.27 (1.22–1.33)	1.51 (1.44–1.58)

*Note*: Model 1 was adjusted for age and sex and Model 2 was adjusted for age, sex, body mass index, income levels, serum alanine transaminase levels, lifestyle factors (smoking status, alcohol consumption, physical activity), hypertension, dyslipidemia, and Charlson Comorbidity Index score. Model 3 was additionally adjusted for presence of T2DM complications and duration of T2DM to covariates in model 2.

Abbreviations: CI, confidence interval; HR, hazard ratio; IR, incidence rate per 1000 person years; T2DM, type 2 diabetes mellitus.

### 3.4 Stratified analysis

**FIGURE 3:**
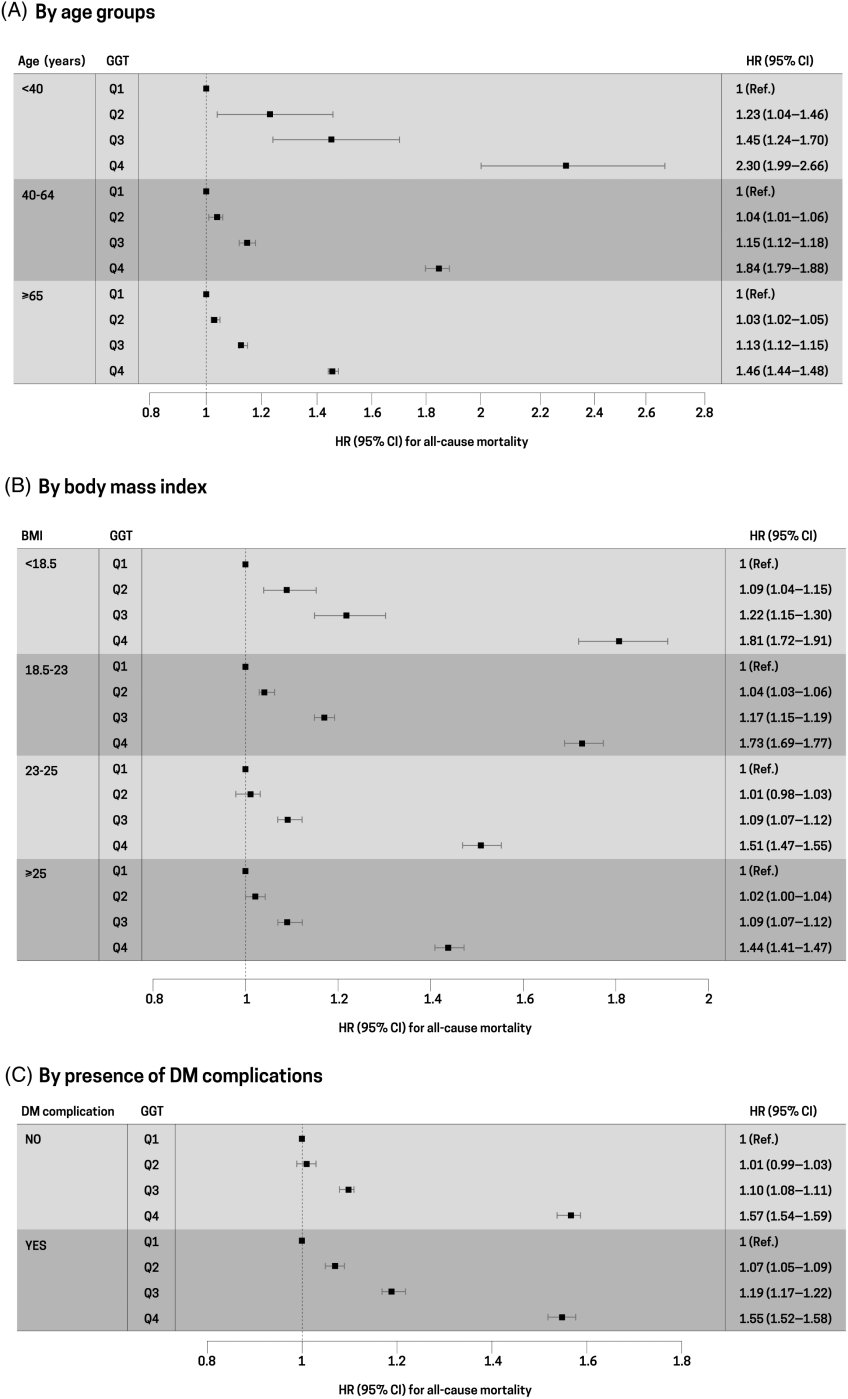
Stratification analysis by (A) age groups, (B) body mass index, and (C) presence of diabetes mellitus complications. HRs are adjusted for age, sex, body mass index, income levels, serum alanine transaminase levels, lifestyle factors (smoking status, alcohol consumption, physical activity), hypertension, dyslipidemia, Charlson comorbidity score, and duration of T2DM. CI, confidence interval; GGT, gamma‐glutamyl transferase; HR, hazard ratio; T2DM, type 2 diabetes mellitus.

Stratified analysis by age, BMI, and presence of T2DM complications was conducted to evaluate the different associations among subgroups. The risk of all‐cause mortality increased as the GGT quartiles became higher across all age groups. The increased risk of all‐cause mortality in young participants aged <40 years (HR, 2.30; 95% CI, 1.99–2.66 in the highest GGT quartile) was more prominent than that in participants aged 40–64 years (HR, 1.84; 95% CI, 1.79–1.88 in the highest GGT quartile) and participants aged ≥65 years (HR, 1.46; 95% CI, 1.44–1.48 in the highest GGT quartile) (*p* for interaction <.001; Figure 3A). Because the agreed cutoff for inclusion in the overweight category is 23.0 kg/m^2^ in Asia‐Pacific countries including Korea, we performed stratified analysis by BMI categories: <18.5, 18.5–23, 23–25, and ≥ 25 kg/m^2^.^28^ Stratified analysis by BMI also showed consistent results—that is, the risk of mortality increased as the GGT quartiles became higher. In the highest GGT quartile, the risk of mortality in underweight participants with BMI <18.5 kg/m^2^ was higher (HR, 1.81; 95% CI, 1.72–1.91) than that in participants with BMI of 18.5–23 kg/m^2^ (HR, 1.73; 95% CI, 1.70–1.77), participants with BMI of 23–25 kg/m^2^ (HR, 1.51; 95% CI, 1.47–1.55), and participants with BMI ≥25 kg/m^2^ (HR, 1.44; 95% CI, 1.41–1.47) (*p* for interaction <.001; Figure 3B). When stratified by the presence of T2DM complications, a stronger association was observed in the group with T2DM complications (HR, 1.57; 95% CI, 1.54–1.59) than in the group without T2DM complications (HR, 1.55; 95% CI, 1.52–1.58) (*p* for interaction <.001; Figure 3C).

In addition, we evaluated the association between GGT quartiles and all‐cause mortality stratified by DM medication (oral hypoglycemic agent or insulin) and duration. The increased risk of all‐cause mortality in participants with DM medication (HR, 1.56; 95% CI, 1.51–1.58 in the highest GGT quartile) was more prominent than that in participants without DM medication (HR, 1.54; 95% CI, 1.51–1.58 in the highest GGT quartile) (*p* for interaction <.001). Also, the risk of all‐cause mortality was increased in the highest GGT quartile across all categories of DM duration (Table S4).

## 4 DISCUSSION

The present study revealed the significant association of serum GGT levels with all‐cause and disease‐specific mortality in patients with T2DM based on a nationwide cohort. GGT levels were positively associated with various causes of death, including CVD, cancer, liver disease, respiratory disease, and infectious disease. Notably, the increased risk of all‐cause mortality was more prominent in the young, underweight, and T2DM complication subgroups, suggesting the usefulness of GGT levels as a prognostic marker in these subgroups.

Previous studies investigated the association between serum GGT levels and mortality in patients with diabetes; however, some of these studies did not specifically exclude patients with liver disease or alcohol abuse, which might have obscured their results.^15^, ^17^ A recent study on community‐dwelling patients with T2DM in China reported that elevated serum GGT concentrations were associated with all‐cause, CVD‐specific, and cancer‐specific mortality. Nevertheless, in this previous study, the rate of current alcohol consumption among participants was 16.9%, which might have influenced the association of serum GGT levels with all‐cause and disease‐specific mortality.^19^ In the present study, we excluded patients with a history of liver disease (eg, viral hepatitis, liver cirrhosis) and heavy drinkers and also adjusted for possible established risk factors in order to explore the robust association between serum GGT levels and mortality.

Previous reports also showed the association between serum GGT levels and CVD‐specific mortality in patients with T2DM.^29^, ^30^, ^31^ In the present study, the risk of both ischemic heart disease and stroke increased in patients with high GGT levels, which is consistent with the findings of a previous study reporting an increased risk of coronary heart disease in patients with T2DM as their serum GGT levels became elevated.^29^ The localization of GGT in coronary atherosclerotic plaques and the involvement of GGT in the oxidation of low‐density lipoprotein cholesterol within diseased vessels imply its role in the progression of atherosclerosis, independent of conventional CVD risk factors and alcohol consumption.^32^

The association between serum GGT activity and mortality from liver disease or cancer has been investigated in the general population.^13^, ^14^ Kazemi‐Shirazi et al showed that serum GGT concentrations above the reference level were significantly associated with cancer‐, hepatobiliary disease‐, and vascular disease‐related mortality in both men and women.^31^ In contrast, studies investigating the association between serum GGT levels and mortality from various diseases in patients with T2DM are limited. A positive association between GGT categories and overall cancer risk was reported, and this association was observed to be strong in patients with elevated glucose levels.^33^, ^34^ In the present study, cancer‐ and liver disease‐related mortality rates increased with increasing GGT levels in patients with T2DM.

The underlying mechanisms by which elevated serum GGT levels contribute to an increased risk of all‐cause and disease‐specific mortality have not been fully understood. Nevertheless, the most obvious explanation is that higher serum GGT concentrations simply reflect the coexistence of underlying known risk factors. In clinical practice, an increase in serum GGT levels is commonly regarded as an indicator of alcohol abuse or liver dysfunction.^35^, ^36^ In this study, serum GGT levels in patients with diabetes were correlated with cardiometabolic risk factors (eg, alcohol consumption, smoking, BMI, blood pressure), which may increase the risk of all‐cause mortality. However, the association between elevated GGT levels and higher all‐cause and disease‐specific mortality rates remained even after adjustment for alcohol consumption, serum ALT levels, and cardiometabolic risk factors. This may confer an excess risk to the expected risk incurred by underlying established risk factors. A possible underlying mechanism linking serum GGT levels to the risk of mortality is the presence of non‐alcoholic fatty liver disease (NAFLD).^37^ GGT not only reflects NAFLD but also correlates with abdominal obesity and glucose intolerance via insulin resistance.^38^, ^39^ Another explanation is that active GGT may play a role in the development of reactive oxygen species.^40^ GGT, a pro‐oxidant with pro‐inflammatory activity, affects the antioxidant capacity of glutathione, which is the most important cellular antioxidant in humans.^8^

In this study, GGT levels exhibited a positive association with respiratory disease‐ and infectious disease‐related mortality. Although there exists less evidence regarding the relationship between GGT levels and respiratory disease‐specific mortality, GGT may enable the identification of individuals at high risk for reduced pulmonary function and/or chronic obstructive pulmonary disease among nonsmokers.^41^ As increased oxidant burden plays an important role in the development of chronic obstructive pulmonary disease, GGT may be related to reduced pulmonary function as an early biomarker of oxidative stress.^42^ With respect to infection‐related mortality, higher serum GGT levels have been reported to increase the burden of subclinical inflammation across metabolic states^43^ and may also be related to infection‐related mortality in the T2DM population.

In this study, higher all‐cause and disease‐specific mortality rates were more pronounced among young participants. Consistent with our results, previous studies reported an age‐attenuating effect on the association between serum GGT levels and CVD.^29^, ^30^, ^31^ Wannamethee et al revealed the strong association between serum GGT activity and CVD‐specific mortality in younger men.^30^ Although the effect of higher rates of hazardous drinking in younger individuals cannot be excluded, a relative decrease in total serum GGT levels has been observed among older individuals with coronary artery disease, as compared with their younger counterparts.^44^ Considering that GGT activity is age dependent and that the upper limit of normal (defined as the 95th percentile of GGT levels) increases up to 70 years of age in men and throughout life in women,^45^, ^46^ differences in risk factors according to GGT levels may be attenuated in the elderly. In the present study, the positive associations of GGT levels with all‐cause, CVD‐specific, cancer‐related, respiratory disease‐related, and liver disease‐related mortality were weaker in elderly participants aged ≥65 years than in younger participants aged <65 years; however, the predictive value of GGT remained significant in both groups, suggesting its utility in risk assessment. Additionally, we found a stronger association between serum GGT levels and mortality in underweight participants (BMI <18.5 kg/m^2^). Because BMI reflects not only body fat mass but also lean mass and does not, therefore, provide information on visceral fat accumulation,^47^ the association between high GGT levels and mortality may suggest a relationship between high GGT levels and metabolically unhealthy underweight in T2DM.

The results of this study should be interpreted in light of its limitations. First, serum GGT levels were measured at a single time only, and we could not obtain information on serial changes in GGT levels. Second, this study may have been affected by misclassification bias. To exclude individuals with insulin‐dependent type 1 diabetes, the ICD‐10 code E10, representing type 1 diabetes, was not included in the study. Although the operational definition of T2DM used in this study has been widely used in previous studies,^23^, ^48^ there is a possibility of the existence of other types of diabetes patients in this study. However, considering the large number of patients analyzed, we suggest that the inclusion of patients with minor forms of diabetes would not have biased the overall conclusions of our study. Third, the Korean NHIS database does not provide glycated hemoglobin and random blood glucose levels, thereby limiting the identification and assessment of the severity of type 2 diabetes. And the operational definition does not fully reflect all patients with T2DM in real‐world settings (ie, patients with early‐stage diabetes or those who did not require treatment). However, the operational definition of T2DM based on population‐based NHIS claims data, including diagnostic codes and prescription code, demonstrated good accuracy, specificity, and reliability when compared to standard reference criteria for T2DM definition.^23^ Fourth, because alcohol consumption was assessed only through self‐reporting, the patients' alcoholic beverage consumption might have been underestimated. There is also the potential for unmeasured confounding factors, such as hepatic steatosis and insulin resistance. Finally, a selection bias might have occurred, as the inclusion criteria for this study were based on participants undergoing health checkups. Further studies are required to validate our findings.

In conclusion, even after adjustment for potential confounders, increased serum GGT levels were associated with higher all‐cause and disease‐specific mortality rates in patients with T2DM. This relationship was more pronounced in young, underweight, and T2DM complication subgroups. Serum GGT levels could serve as a relevant parameter for the identification of high‐risk patients with T2DM requiring close monitoring and specific interventions to manage mortality.

## AUTHOR CONTRIBUTIONS

The corresponding authors (Kyungdo Han and Eun Ju Cho) had full access to all the data in the study and takes responsibility for the integrity of the data and the accuracy of the data analysis. Study concept and design: Kyungdo Han, Eun Ju Cho. Provision of study materials or patients: Goh Eun Chung, Su‐Min Jeong, Su Jong Yu, Jeong‐Ju Yoo, Goh Eun Chung, Yuri Cho, Dong Wook Shin, Yun Joon Kim, Jung‐Hwan Yoon, Eun Ju Cho. Collection and assembly of data: Kyu Na Lee, Kyungdo Han. Data analysis and interpretation: Kyu Na Lee, Eun Ju Cho, Su‐Min Jeong, Kyungdo Han, Su Jong Yu. Manuscript writing: Goh Eun Chung, Su‐Min Jeong. Final approval of manuscript: All authors.

## FUNDING INFORMATION

This work was supported by grants from the Seoul National University Hospital Research Fund (04–2022‐0790) and from Liver Research Foundation of Korea as part of the Bio Future Strategies Research Project.

## CONFLICT OF INTEREST STATEMENT

The authors declare no potential conflicts of interest relevant to this manuscript.

## Supporting information


**DATA S1:** Supporting Information.

## Data Availability

The data that support the findings of this study are available from the National Health Insurance Sharing Service (NHISS) of South Korea, but restrictions apply to the availability of these data, which were used under license for the current study, and so are not publicly available. Data are, however, available from the authors upon reasonable request and with permission of the NHISS of South Korea.
