# Supplementary material for: Gamma‐glutamyl transferase and the risk of all‐cause and disease‐specific mortality in patients with diabetes: A nationwide cohort study
Source: J Diabetes. 2024 Apr 25;16(5):e13551. doi: 10.1111/1753-0407.13551 (PMC11045922; doi:10.1111/1753-0407.13551)
Supplement: Supplementary file 1 — DATA S1: Supporting Information. [file JDB-16-e13551-s001.docx]

**Supplementary Table 1.** Information of missing data

| Variables | Number |
| --- | --- |
| Income | 51,403 |
| BMI, WC | 1,866 |
| Systolic/diastolic BP | 695 |
| Cholesterol (Total cholesterol, HDL-C, LDL-C, TG) | 25,223 |
| AST, ALT, GGP | 2,376 |
| Lifestyle (smoking, drinking, regular exercise) | 16,940 |

BMI, body mass index; WC, waist circumference; BP, blood pressure; HDL-C high-density lipoprotein-cholesterol; LDL-C, low-density lipoprotein-cholesterol; AST, aspartate aminotransferase; ALT, alanine transaminase; GGT, gamma-glutamyl Transferase

**Supplementary Table 2.** The association between GGT quartiles and all-cause and CVD-specific mortality by sex

|  |  | Event | Duration | IR, per 1000 | Model1 | Model2 | Model3 |
| --- | --- | --- | --- | --- | --- | --- | --- |
| Male | |  |  |  | HR (95% CI) | HR (95% CI) | HR (95% CI) |
| All-Cause mortality | |  |  |  |  |  |  |
| Q1 | 280,730 | 43,020 | 2,136,802.9 | 20.1 | 1(Ref.) | 1(Ref.) | 1(Ref.) |
| Q2 | 294,293 | 34,614 | 2,277,532.2 | 15.2 | 0.96 (0.94-0.97) | 1.06 (1.04-1.07) | 1.07 (1.06-1.09) |
| Q3 | 284,197 | 26,799 | 2,211,275.9 | 12.1 | 0.98 (0.97-0.99) | 1.16 (1.14-1.18) | 1.18 (1.17-1.20) |
| Q4 | 286,705 | 29,210 | 2,198,027.5 | 13.3 | 1.39 (1.37-1.42) | 1.69 (1.66-1.71) | 1.74 (1.71-1.77) |
| CVD-specific mortality | | | |  |  |  |  |
| Q1 | 280,730 | 8,500 | 2,136,802.9 | 4.0 | 1(Ref.) | 1(Ref.) | 1(Ref.) |
| Q2 | 294,293 | 7,074 | 2,277,532.2 | 3.1 | 1.00 (0.97-1.04) | 1.10 (1.06-1.13) | 1.12 (1.085,1.16) |
| Q3 | 284,197 | 5,336 | 2,211,275.9 | 2.4 | 1.02 (0.98-1.05) | 1.20 (1.15-1.24) | 1.24 (1.198,1.29) |
| Q4 | 286,705 | 4,950 | 2,198,027.5 | 2.3 | 1.25 (1.20-1.29) | 1.58 (1.52-1.64) | 1.67 (1.602,1.74) |
| Ischemic Heart Disease mortality | | | |  |  |  |  |
| Q1 | 280,730 | 2,700 | 2,136,802.9 | 1.3 | 1(Ref.) | 1(Ref.) | 1(Ref.) |
| Q2 | 294,293 | 2,349 | 2,277,532.2 | 1.0 | 1.01 (0.96-1.07) | 1.08 (1.02-1.14) | 1.12 (1.06-1.19) |
| Q3 | 284,197 | 1,782 | 2,211,275.9 | 0.8 | 0.99 (0.93-1.05) | 1.13 (1.06-1.21) | 1.21 (1.14-1.29) |
| Q4 | 286,705 | 1,439 | 2,198,027.5 | 0.7 | 1.02 (0.95-1.09) | 1.25 (1.16-1.34) | 1.38 (1.28-1.49) |
| Stroke mortality | | | |  |  |  |  |
| Q1 | 280,730 | 1,351 | 2,136,802.9 | 0.6 | 1(Ref.) | 1(Ref.) | 1(Ref.) |
| Q2 | 294,293 | 1,054 | 2,277,532.2 | 0.5 | 1.01 (0.93-1.09) | 1.13 (1.04-1.23) | 1.15 (1.06-1.25) |
| Q3 | 284,197 | 798 | 2,211,275.9 | 0.4 | 1.08 (0.99-1.18) | 1.34 (1.22-1.47) | 1.38 (1.26-1.51) |
| Q4 | 286,705 | 710 | 2,198,027.5 | 0.3 | 1.35 (1.23-1.48) | 1.84 (1.66-2.04) | 1.91 (1.72-2.12) |
| Female | |  |  |  | HR (95% CI) | HR (95% CI) | HR (95% CI) |
| All-Cause mortality | |  |  |  |  |  |  |
| Q1 | 225,522 | 26,535 | 1,759,504.4 | 15.1 | 1(Ref.) | 1(Ref.) | 1(Ref.) |
| Q2 | 199,871 | 20,611 | 1,577,597.9 | 13.1 | 0.91 (0.89-0.93) | 0.96 (0.94-0.97) | 0.96 (0.95-0.98) |
| Q3 | 224,010 | 21,295 | 1,771,673.6 | 12.2 | 0.93 (0.91-0.95) | 1.02 (1.00-1.04) | 1.04 (1.02-1.06) |
| Q4 | 212,447 | 20,158 | 1,662,380.6 | 12.1 | 1.12 (1.10-1.14) | 1.31 (1.29-1.34) | 1.35 (1.32-1.38) |
| CVD-specific mortality | | | |  |  |  |  |
| Q1 | 225,522 | 6,443 | 1,759,504.4 | 3.7 | 1(Ref.) | 1(Ref.) | 1(Ref.) |
| Q2 | 199,871 | 5,114 | 1,577,597.9 | 3.2 | 0.94 (0.91-0.98) | 0.99 (0.95-1.03) | 0.99 (0.96-1.04) |
| Q3 | 224,010 | 5,255 | 1,771,673.6 | 3.0 | 0.97 (0.94-1.01) | 1.08 (1.04-1.12) | 1.10 (1.05-1.14) |
| Q4 | 212,447 | 4,896 | 1662380.56 | 2.9 | 1.17 (1.13-1.22) | 1.44 (1.38-1.50) | 1.48 (1.42-1.55) |
| Ischemic Heart Disease mortality | | | |  |  |  |  |
| Q1 | 225,522 | 1,549 | 1,759,504.4 | 0.9 | 1(Ref.) | 1(Ref.) | 1(Ref.) |
| Q2 | 199,871 | 1,303 | 1,577,597.9 | 0.8 | 0.99 (0.92-1.07) | 1.03 (0.96-1.11) | 1.05 (0.97-1.13) |
| Q3 | 224,010 | 1,342 | 1,771,673.6 | 0.8 | 1.02 (0.95-1.10) | 1.11 (1.03-1.20) | 1.15 (1.07-1.24) |
| Q4 | 212,447 | 1,144 | 1,662,380.6 | 0.7 | 1.11 (1.03-1.20) | 1.38 (1.27-1.50) | 1.46 (1.34-1.59) |
| Stroke mortality | | | |  |  |  |  |
| Q1 | 225,522 | 971 | 1,759,504.4 | 0.6 | 1(Ref.) | 1(Ref.) | 1(Ref.) |
| Q2 | 199,871 | 764 | 1,577,597.9 | 0.5 | 0.95 (0.87-1.05) | 0.99 (0.90-1.09) | 1.00 (0.91-1.10) |
| Q3 | 224,010 | 798 | 1,771,673.6 | 0.5 | 1.01 (0.92-1.11) | 1.10 (1.00-1.22) | 1.12 (1.01-1.23) |
| Q4 | 212,447 | 767 | 1,662,380.6 | 0.5 | 1.27 (1.15-1.39) | 1.48 (1.33-1.64) | 1.51 (1.36-1.68) |

IR, incidence rate per 1000 person years; HR, hazard ratio; CI, confidence intervals; CVD, cardiovascular disease; T2DM, type 2 diabetes mellitus

Model 1 was adjusted for age and sex and Model 2 was adjusted for age, sex, body mass index, income levels, serum alanine transaminase levels, lifestyle factors (smoking status, alcohol consumption, physical activity), hypertension, dyslipidemia, and CCI score. Model 3 was additionally adjusted for presence of T2DM complications and duration of T2DM to covariates in model 2.

**Supplementary Table 3.** Sensitivity analysis: The association between gamma-glutamyl transferase quartiles and all-cause mortality.

|  | GGT quartile | Number | No. of Event | Duration | IR, per 1000 | HR (95% CI) |
| --- | --- | --- | --- | --- | --- | --- |
| No CVD | Q1 | 437,470 | 54,160 | 3386700.3 | 16.0 | 1(Ref.) |
|  | Q2 | 428,177 | 42,523 | 3356682.5 | 12.7 | 1.02 (1.01,1.03) |
|  | Q3 | 444,561 | 37,072 | 3497615.2 | 10.6 | 1.11 (1.10,1.13) |
|  | Q4 | 445,332 | 39,807 | 3455103.1 | 11.5 | 1.58 (1.55,1.60) |
| No cancer | Q1 | 488,777 | 64,588 | 3776100.0 | 17.1 | 1(Ref.) |
|  | Q2 | 480,009 | 51,567 | 3755964.4 | 13.7 | 1.02 (1.01,1.04) |
|  | Q3 | 495,734 | 45,136 | 3894867.3 | 11.6 | 1.12 (1.10,1.13) |
|  | Q4 | 488,324 | 46,439 | 3787107.1 | 12.3 | 1.55 (1.53,1.57) |

IR, incidence rate per 1000 person years; HR, hazard ratio; CI, confidence intervals; CVD, cardiovascular disease

Multivariable model was adjusted for age, sex, body mass index, income levels, serum alanine transaminase levels, lifestyle factors (smoking status, alcohol consumption, physical activity), hypertension, dyslipidemia, CCI score, presence of T2DM complications and duration of T2DM.

**Supplementary Table 4.** The association between gamma-glutamyl transferase quartiles and all-cause mortality by DM medication and duration.

|  | GGT quartile | | Number | | No. of Event | | Duration | | IR, per 1000 | | HR (95% CI) | |
| --- | --- | --- | --- | --- | --- | --- | --- | --- | --- | --- | --- | --- |
| DM medication | | |  | |  | |  | |  | |  | |
|  | Q1 | | 168,506 | | 15,051 | | 1291306.8 | | 11.7 | | 1 (Ref.) | |
| No | Q2 | | 178,645 | | 12,436 | | 1385054.4 | | 8.0 | | 0.97 (0.94, 0.99) | |
|  | Q3 | | 202,633 | | 11,754 | | 1575915.7 | | 7.5 | | 1.04 (1.02, 1.07) | |
|  | Q4 | | 232,754 | | 15,413 | | 1791415.3 | | 8.6 | | 1.54 (1.51, 1.58) | |
|  | Q1 | | 337,746 | | 54,504 | | 2605000.7 | | 20.9 | | 1 (Ref.) | |
| Yes | Q2 | | 315,519 | | 42,789 | | 2470075.6 | | 17.3 | | 1.05 (1.03, 1.06) | |
|  | Q3 | | 305,574 | | 36,340 | | 2407033.9 | | 15.1 | | 1.15 (1.14, 1.17) | |
|  | Q4 | | 266,398 | | 33,955 | | 2068992.8 | | 16.4 | | 1.56 (1.54, 1.59) | |
| DM duration | |  | |  | |  | |  | |  | |  |
|  | Q1 | 160,132 | | 13,582 | | 1229462.4 | | 11.0 | | 1 (Ref.) | |  |
| New onset | Q2 | 170,287 | | 11,335 | | 1321813.3 | | 8.6 | | 0.97 (0.94, 0.99) | |  |
|  | Q3 | 194,829 | | 10,827 | | 1516594.8 | | 7.1 | | 1.04 (1.02, 1.07) | |  |
|  | Q4 | 225,971 | | 14,501 | | 1740910.5 | | 8.3 | | 1.56 (1.52, 1.60) | |  |
|  | Q1 | 140,198 | | 18,045 | | 1094703.8 | | 16.5 | | 1 (Ref.) | |  |
| < 5 years | Q2 | 154,395 | | 16,873 | | 1220181.6 | | 13.8 | | 1.04 (1.02, 1.06) | |  |
|  | Q3 | 169,618 | | 16,018 | | 1346544.7 | | 11.9 | | 1.12 (1.10, 1.15) | |  |
|  | Q4 | 166,327 | | 17,174 | | 1303301.7 | | 13.2 | | 1.57 (1.54, 1.61) | |  |
|  | Q1 | 205,922 | | 37,928 | | 1572141.1 | | 24.1 | | 1 (Ref.) | |  |
| ≥5 years | Q2 | 169,482 | | 27,017 | | 1313135.2 | | 20.6 | | 1.05 (1.03, 1.07) | |  |
|  | Q3 | 143,760 | | 21,249 | | 1119810.1 | | 19.0 | | 1.17 (1.15, 1.19) | |  |
|  | Q4 | 106,854 | | 17,693 | | 816196.0 | | 21.7 | | 1.54 (1.51, 1.57) | |  |

IR, incidence rate per 1000 person years; HR, hazard ratio; CI, confidence intervals; CVD, cardiovascular disease; DM, diabetes mellitus

Multivariable model was adjusted for age, sex, body mass index, income levels, serum alanine transaminase levels, lifestyle factors (smoking status, alcohol consumption, physical activity), hypertension, dyslipidemia, CCI score, presence of T2DM complications and duration of T2DM.
